# Mineral and Bone Disease in Black African Hemodialysis Patients: A Report From Senegal

**DOI:** 10.5812/numonthly.4225

**Published:** 2012-09-24

**Authors:** Sidy Mohamed Seck, Mohamed Dahaba, Elhadj Fary Ka, Mouhamadou Moustapha Cisse, Seigne Gueye, Ahmet Ould Lemrabott Tal

**Affiliations:** 1 Internal Medicine and Nephrology Department, Faculty of Health Sciences, University Gaston Berger, Saint-Louis, Senegal; 2 Hemodialysis Unit, Polyclinic ABC, Dakar, Senegal; 3 Nephrology Department, University Hospital Aristide Le Dantec, Dakar, Senegal

**Keywords:** Renal Osteodystrophy, Hemodialysis, Senegal

## Abstract

**Background:**

Chronic kidney disease related mineral and bone disease (CKD-MBD) is a worldwide challenge in hemodialysis patients. In Senegal, number of dialysis patients is growing but few data are available about their bone disorders.

**Objectives:**

To describe patterns of CKD-MBD in Senegalese dialysis patients.

**Patients and Methods:**

We performed a cross-sectional study including patients from three dialysis centres in Senegal. Diagnosis of different types of CKD-MBD relied on clinical, biological and radiological data collected from medical records in dialysis.

**Results:**

We included 118 patients and 79 of them presented CKD-BMD (prevalence of was 66.9 %). Mean age of CKD-MBD patients was 47.8 ± 15.7 years (16-81 years) and sex-ratio (Male/Female) was 1.15. Secondary hyperparathyroidism was the most frequent disorder (57 patients) followed by adynamic bone disease (21 patients) and osteomalacia (1 patients). The main clinical manifestations were bone pain (17.5% of cases), pruritus (36.8% of cases) and pathological fractures (2.5% of cases). Bone biopsy was not available. Valvular and peripheral vascular calcification were present in 24.5% and 21.2% of patients respectively. Management of CKD-MBD included optimization of dialysis, calcium bicarbonate, sevelamer, vitamin D analogues and calcimimetics. The NKF/DOQI recommended levels of serum calcium, phosphate and parathormone PTH were not achieved in one third of patients. Six patients presented major cardiovascular events during their dialysis period.

**Conclusions:**

CKD-MBD are frequent in Senegalese hemodialysis patients and they are dominated by high turn-over disease. Clinical and biological manifestations are unspecific and accurate diagnoses are often difficult in absence of histomorphometry. Treatment is suboptimal for many patients in a context of limited resources.

## 1. Background

Chronic kidney disease related mineral and bone disease (CKD-MBD) previously called renal osteodystrophy is frequent in hemodialysis patients and may increase morbidity and cardiovascular mortality of patients (1, 2). Almost all patients starting hemodialysis presents mineral disorders and bone modifications which took place since CKD stage 3-5 (3). Moreover, racial differences were found in American patients undergoing dialysis (2). In Africa, despite a growing number of patients entering hemodialysis, data about CKD-MBD are very rare and limited to small-sized sample of patients (4). The objective of this study was to describe epidemiological patterns of CKD-MBD in end-stage renal disease (ESRD) patients undergoing chronic hemodialysis in Senegal.

## 2. Objectives

The aim of this study was to describe epidemiological patterns of CKD-MBD in end-stage renal disease patients undergoing chronic hemodialysis in Senegal.

## 3. Patients and Methods

We performed a cross-sectional study in three dialysis centres in Senegal between September and November 2011. All ESRD patients hemodialysed since at least three months and with up-to-date biochemical explorations. Patients whose laboratory explorations were incomplete or older than six months were not included. For each patient, we collected the following information from medical records: age, gender, health coverage, clinical symptoms, serum level of calcium, phosphorus, intact parathormone (iPTH), alkaline phosphatase, aluminum, C-reactive protein (CRP), hemoglobin and 25(OH)vitamin D (calcidiol). Laboratory parameters were measured quarterly except for iPTH, calcidiol, aluminemia and CRP which were dosed every six months. Cardiovascular calcifications and atherosclerosis were assessed by echocardiography and Doppler exploration of peripheral vessels. Dialysis parameters (dialysis vintage, weekly number and duration of sessions, dialysate calcium concentration and interdialytic weight gain) and ongoing medications were also recorded.

In absence of bone histology, diagnosis of CKD-MBD subtypes relied on the association of clinical and biological findings and management of patients followed KDIGO guidelines (3). According to their iPTH levels, patients were classified in high turn-over osteopathy (hyperparathyroidism) or low turn-over osteopathy (adynamic bone disease or osteomalacia). Osteomalacia was retained if there was a low iPTH level and typical Looser zones on standard X-ray. Statistical analyses were performed using Microsoft Excel 2007 and SPSS 16.0 (Chicago, IL). Data were presented as mean ± standard deviation or percentage according to the type of variable. To compare means or percentage we used t-test, Man-Whitney, Chi-square tests as appropriate. Risk factors associated with CKD-MBD were identified using a logistic regression analysis. All results were considered statistically significant when value was ≤ 0.05.

## 4. Results

**Figure 1:**
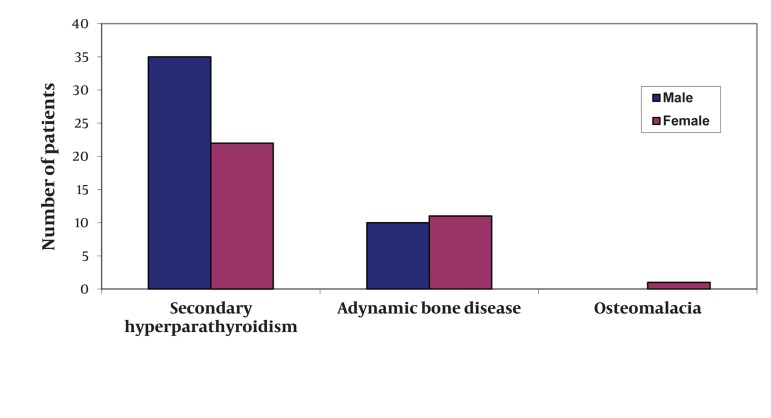
CKD-MBD Subtypes According to Gender

During the study period, we included 118 patients and 79 of them presented CKD-BMD (prevalence of 66.9 %). Mean age of CKD-MBD patients was 47.8 ± 15.7 years (16-81 years) and sex-ratio (Male/Female) was 1.15 (Figure 1). The predominant cause of end-stage renal disease was hypertension (44.0%), followed by chronic glomerulonephritis (12.5%), diabetes (7.5%), autosomal dominant polycystic kidney disease (7.0%) and vasculitis (5%). In 24.0% of patients, the etiology was unknown. The median hemodialysis vintage was 45 ± 30.5 months (03-132 months) and median weekly dialysis time was 10 hours (8-12 hours). All patients were dialysed with a 1.75 mmol/L calcium dialysate and mean interdialytic weight gain was 3.28 kg (0.5-6 kg). CKD-MBD subtypes regrouped 57 cases of secondary hyperparathyroidism (SHPT), 21 with adynamic bone disease (ABD) and 1 case with radiological findings compatible with osteomalacia (OM). Clinical and biological characteristics of patients are summarized in Table 1. Bone biopsy was not performed because it was not available in our country. Valvular and peripheral vascular calcification were present respectively in 24.5% and 21.2% of patients. Only calcemia was associated with serum level of iPTH (r = -0.34, P = 0.03). Vascular calcifications were directly correlated with calcemia (r = 0.86; P = 0.02) and cardiac valvular calcifications with iPTH level (r = 0.53; P = 0,01). Logistic regression analysis did not find any association between CKD-MBD subtype and age, gender, dialysis vintage or KT/V. Management of patients with SHPT included optimization of dialysis parameters to achieve a KT/V ≥ 1.2 dose (for all patients) in combination with prescription of calcium bicarbonate (in 26 patients), sevelamer hydrochloride (in 02 patients), 1-alphacalcidol (in 30 patients), calcitriol (in 04 patients) and cinacalcet (in 02 patients). In patients with low turn-over osteodystrophy, treatment comprised also dialysis optimization, calcium bicarbonate (in 09 patients), and 1-alphacalcidol (in 08 patients). Subtotal parathyroidectomia was performed in three patients with refractory SHPT. Despite all these therapies, levels of serum calcium, phosphate and iPTH recommended by NKF/DOQI guidelines were not achieved in 31.6%, 38.6% and 35.0% of patients respectively. Six patients had presented myocardial infarction and/or stroke during their dialysis period.

**Table 1:** Clinical and Biological Characteristics of Patients With CKD Related Mineral and Bone Disease (mean ± standard deviation and extremes)

	High Turn Over disease, (n = 57)	Low Turn Over Disease, (n = 22)
**Age (y)**	46.4 ± 25.7 (16-76)	50.2 ± 38.1 (31-81)
**Sex-ratio (M/F)**	1.6	1.2
**Dialysis vintage (mon)**	53.6 ± 9.3	78.4 ± 5.9
**Weekly KT/V**	1.3	1.4
**Clinical symptoms (NO. of cases)**		
Bone pain	25	17
Pruritus	14	09
Bone fractures	01	02
**Biological parameters**		
Calcemia (mg/dL)	8.6 ± 6.3 (5.7-9.8)	9.5 ± 7.1 (8.8-11.7)
Phosphatemia (mg/L)	4.85 ± 3.1 (3.5-13.8)	3.2 ± 1.0 (1.4-10.5)
Product CaxP (mg^2^/dL^2^)	98.8 ± 20.2 (18.0-123.3)	34.5 ± 10.3 (16.4-95.7)
Alkaline phosphatases (UI/L)	250.5 ± 123.8 (142-654)	120 ± 74.7 (86-290)
Parathormone intact (ng/L)	984.2 ± 146.8 (196-1385)	60.5 ± 56.3 (27-148)
Hemoglobin (g/dL)	9.1 ± 8.3 (5.7-16.6)	8.7 ± 5.6 (6.4-11.8)
C reactive protein (mg/L)	12.7 ± 4.3 (0-96)	6.4 ± 3.5 (0-48)

## 5. Discussion

Data about the burden of CKD-MBD in African dialysis populations are very scarce. Our results showed similar high prevalence like in previous reports from western and African countries (1, 2, 5).

In comparison with a previous study in one Senegalese dialysis unit, the prevalence of SHPT is increasing (from 31% in 2004 to 48.3% in this study) (4). SHPT was found in 11.8% of Nigerian patients with ESRD (6). In African Americans hemodialysis patients with bone histology, SHPT is far more frequent than low turn-over osteopathy (2, 7).

Retrospective series of bone biopsies from Brazil and Uruguay noticed the same rising trend of SHPT contrasting with a sharp drop of aluminum poisoning (8). High frequency of CKD-MBD in our patients can be explained by a poor control of phosphate balance due to difficult access to hemodialysis and expensive drugs therapies. Of note, the majority of our patients had no health insurance and they were dialysed less than 12 hours a week Lower prevalence of hyperparathyroidism was reported in Libya but adynamic bone disease was more frequent than in our patients (5). In our patients aluminum toxicity was not described in absence of bone biopsy but it might be low as phosphate binders containing aluminum were exceptionally prescribed. Also, low turn-over osteodystrophy in our study was less frequent than reported in Canada (9). This can be related to a younger age of our patients like in many developing countries where access to dialysis is generally limited to active adult population (10). Manifestations of CKD-MBD were unspecific and the majority of our patients were asymptomatic. This lack of specificity of clinical symptoms had been emphasized by many authors and it makes diagnosis of CKD-MBD more difficult (10-12). In the study by Hercz et al., about two thirds of patients with aplastic osteopathy did not present any clinical symptom (9). Radiological bone modifications were not explored in our study because of their low diagnostic value in patients with suspected CKD-MBD (12, 13). Plain X-ray can help detect vascular calcifications which were associated with higher incidence of cardiovascular events (14). In this study, only iPTH was correlated valvular calcifications and calcifications in peripheral vessels were associated with calcemia. Pathophysiology of vascular calcifications in hemodialysis patients is complex and many risk factors such as age, male sex, diabetes and FGF-23 were identified (14, 15). Hypocalcemia was frequent in our patients with high turn-over osteodystrophy and it represented with hyperphosphatemia and vitamin D deficiency the main stimulating factors responsible for parathyroid hyperactivity. In an Italian study, 35.5% of hemodialysis patients presented a product Ca x P > 55 mg^2^/dL^2^ and the mean iPTH level was 318 ± 413 ng/L (16). This study presents many limits due to its cross-sectional nature and absence of histomorphometry which makes difficult accurate diagnosis of different CKD-MBD subtypes (3, 11). Also, interpretation of parathormone levels in black subjects might be careful because many cases of ABD were demonstrated in black patients with iPTH within the normal ranges (17, 18). High levels of iPTH is not synonymous of bone fibrosis. Some authors supported hypothesis of “a bone resistance to parathormone effects” in blacks who can tolerate high parathormone levels without significant bone remodeling (19, 20). Recent therapies such as sevelamer, lanthanum and calcimimetics have demonstrated high efficiency in patients with SHP (21, 22). However, their onerous cost was inaccessible for the majority of our patients. In ABD, the use of vitamin D analogues was limited by the risk of hypercalcemia (22). In patients with severe SHPT, supplementation with 25 (OH) vitamin D might have specific effects independent of those of calcitriol (23) but the relevance of such attitude needs to be demonstrated in our context of tropical sunny countries. Despite poor phosphate control in one third of our patients, parathyroidectomy was rarely performed and cardiovascular mortality was not as high as reported by other studies (24). In African Americans, only 9.5% presented simultaneously serum calcium, phosphate and iPTH levels within the recommended K/DOQI ranges (7).

CKD-MBD is a common disease in hemodialysis patients in Senegal. It is dominated by high turn-over osteodystrophy but other types might be under-diagnosed because of the absence of bone histology. Clinical and biological findings are not specific and accurate diagnosis is limited by absence of bone histology. Extra-skeletal calcification were not rare and they correlated with calcium status. Treatment associated dialysis optimization and drug therapy that are often unavailable in our context.
